# Predictive value of a false-negative focused abdominal sonography for trauma (FAST) result in patients with confirmed traumatic abdominal injury

**DOI:** 10.1186/s13244-020-00911-5

**Published:** 2020-09-23

**Authors:** Mohammed H. A. Alramdan, Derya Yakar, Frank F. A. IJpma, Ömer Kasalak, Thomas C. Kwee

**Affiliations:** 1 Medical Imaging Center, Department of Radiology, Nuclear Medicine and Molecular Imaging, University of Groningen, University Medical Center Groningen, Hanzeplein 1, P.O. Box 30.001, 9700 RB Groningen, The Netherlands; 2 Department of Trauma Surgery, University of Groningen, University Medical Center Groningen, Groningen, The Netherlands

**Keywords:** Abdomen, FAST exam, Patient outcome assessment, Trauma, Ultrasonography

## Abstract

**Objective:**

To investigate if patients with confirmed traumatic abdominal injury and a false-negative focused abdominal sonography for trauma (FAST) examination have a more favorable prognosis than those with a true-positive FAST.

**Methods:**

This study included 97 consecutive patients with confirmed traumatic abdominal injury (based on computed tomography [CT] and/or surgical findings) who underwent FAST.

**Results:**

FAST was false-negative in 40 patients (41.2%) and true-positive in 57 patients (58.8%). Twenty-two patients (22.7%) had an unfavorable outcome (defined as the need for an interventional radiologic procedure, laparotomy, or death due to abdominal injury). Univariately, a false-negative FAST (odds ratio [OR] 0.24; *p* = 0.017) and a higher systolic blood pressure (OR, 0.97 per mmHg increase; *p* = 0.034) were significantly associated with a favorable outcome, whereas contrast extravasation on CT (OR, 7.17; *p* = 0.001) and shock index classification (OR, 1.89 for each higher class; *p* = 0.046) were significantly associated with an unfavorable outcome. Multivariately, only contrast extravasation on CT remained significantly associated with an unfavorable outcome (OR, 4.64; *p* = 0.016). When excluding contrast extravasation on CT from multivariate analysis, only a false-negative FAST result was predictive of a favorable outcome (OR, 0.28; *p* = 0.038).

**Conclusion:**

Trauma patients with confirmed abdominal injury and a false-negative FAST have a better outcome than those with a positive FAST. FAST may be valuable for risk stratification and prognostication in patients with a high suspicion of abdominal injury when CT has not been performed yet or when CT is not available.

## Key points

- A false-negative focused abdominal sonography for trauma (FAST) in confirmed traumatic abdominal injury is associated with a more favorable outcome.
- On a group level, the FAST result is superior to basic vital parameters (including systolic blood pressure and shock index classification), but not to contrast extravasation status on computed tomography (CT), in predicting outcome in confirmed traumatic abdominal injury.
- FAST may be valuable for risk stratification and prognostication in patients with a high suspicion of abdominal injury when CT has not been performed yet or when CT is not available.

## Introduction

Injury is an important cause of morbidity and mortality in the developed and developing world [1]. Up to 20% of severe trauma patients are diagnosed with severe abdominal injury, and this is associated with a mortality rate of around 20% [2]. Concealed hemorrhage is a major cause of death after trauma, and missed abdominal injuries are a frequent cause of morbidity and late mortality in patients who survive the early period after injury [3]. Imaging plays an important role in patients with suspected abdominal injury [4].

Ultrasonography is a non-invasive imaging modality that does not use any ionizing radiation, and can be performed in the emergency department simultaneously with other aspects of resuscitation [1]. Focused abdominal sonography for trauma (FAST) is an abbreviated, protocolized form of ultrasonography to rapidly screen for the presence of free intra-abdominal fluid as an indirect sign of abdominal injury [1]. However, a normal FAST does not exclude injury [3]. A meta-analysis that investigated the value of FAST for the detection of free intra-abdominal fluid in a total of 19,666 trauma patients reported a pooled sensitivity of 74.2% and a pooled specificity of 97.6% [4]. These data underline that FAST in trauma is helpful to rule in, but not to rule out, free intra-abdominal fluid [4].

Meanwhile, computed tomography (CT) is considered the imaging modality of choice for the evaluation of hemodynamically stable patients [1]. Early repeated CT in initially non-operated patients with blunt bowel and mesenteric injuries has also been reported to improve the sensitivity of lesion detection requiring surgical repair and the security of patient selection for nonoperative treatment [5]. However, a disadvantage of CT is the use of potentially harmful ionizing radiation and intravenous contrast agents [1]. Nevertheless, with the use of optimized protocols (such as biphasic CT with iterative reconstruction techniques [6]) and appropriate selection criteria for CT scanning [7], these issues can be somewhat mitigated. Another potential disadvantage of CT is that the patient may have to be moved away from the resuscitation area, as a result of which it is generally not appropriate for the evaluation of hemodynamically instable patients [3]. Because of these issues, and the fact that the use of FAST is endorsed by the European guidelines on the treatment of patients with severe/multiple injuries [8], FAST still has a routine role in the evaluation of trauma patients in many institutions, including ours.

Although there is an abundance of literature on the diagnostic value of FAST [4], and its suboptimal sensitivity is widely recognized (in part dependent on the training of the FAST operator and the volume of free intra-peritoneal fluid [9], if any [10]) [3, 4], the value of a false-negative FAST (i.e., a negative FAST in a patient who actually has abdominal injury) in predicting outcome remains unclear. It is hypothesized that a false-negative FAST reflects a lower risk of (hemodynamically) significant organ damage than a true positive FAST, and that the outcome of the former group will be more favorable than the latter in terms of the need for a subsequent interventional radiologic procedure or laparotomy, and death. Such information may provide evidence-based data to determine whether abdominal CT is still necessary after negative FAST, to establish the utility of FAST in triaging patients when staff and CT availability are (temporarily) limited, and to demonstrate the value of FAST as a stand-alone tool when CT is not available. It may also help to determine which cases require more intense observation, and it can be useful to inform patients about their prognosis. A previous study reported patients with abdominal injury and a false-negative FAST result to be substantially less likely to require therapeutic laparotomy with an odds ratio [OR] of 0.31 (95% confidence interval [CI], 0.19–0.52) [11]. Although these results support our hypothesis, more research is necessary to confirm these preliminary results.

The purpose of this study was to investigate if patients with confirmed traumatic abdominal injury and a false-negative FAST have a more favorable prognosis than those with a true-positive FAST. Note that traumatic abdominal injury was defined as organ injury or pathologic free fluid in the intra- or retroperitoneal space, and that a negative FAST result in a patient with retroperitoneal injury was also considered “false-negative” for the purpose of this study.

## Materials and methods

### Study design

This study was approved by the local institutional review board and the requirement for informed consent was waived. The University Medical Center Groningen is a tertiary care level 1 trauma center in the north-east of The Netherlands. Any trauma patient with a suspicion of abdominal injury or in whom abdominal injury cannot be ruled out prior to arrival in the emergency room of the hospital undergoes a FAST examination. All trauma patients who underwent FAST between January 2018 and February 2020 were potentially eligible for inclusion. Patients were included if they had confirmed organ injury or pathologic free fluid in the intra- or retroperitoneal space on CT or surgery. Patients with a true-negative FAST (i.e., negative FAST without organ injury or pathologic free fluid in the intra- or retroperitoneal space on CT or surgery), patients with a false-positive FAST (i.e., positive FAST without organ injury or pathologic free fluid in the intra- or retroperitoneal space on CT or surgery), patients with an inconclusive FAST, and patients with free intra-abdominal fluid on FAST that could be due to pre-existing conditions as recorded in their medical files and known to the healthcare providers upon arrival of the patient in the hospital [12] were excluded.

### Clinical variables

Medical records were reviewed to retrieve patient age and gender, mechanism of injury, whether code red was assigned (indicating the highest level of urgency and possible major hemorrhage [13]), basic vital parameters upon arrival in the hospital including hemoglobin level (subsequently classified as low or normal/high according to local reference values), heart rate, systolic blood pressure, shock index (i.e., ratio between heart rate and systolic blood pressure), and shock index class (class 1 [no shock]: shock index < 0.6; class 2 [mild shock]: shock index ≥ 0.6 to < 1.0; class 3 [moderate shock]: shock index ≥ 1.0 to < 1.4; and class 4 [severe shock]: shock index ≥ 1.4 [14]), presence of extra-abdominal injury, time between FAST and abdominal CT (if performed), and hospitalization days. In addition to these clinical variables, the need for an interventional radiologic procedure, laparotomy, or death due to abdominal injury was also extracted from the medical records.

### FAST

All FAST examinations were performed by 27 different radiology residents upon arrival of the patient in the emergency room of the hospital, either independently after formal training and an assigned entrustable professional activity to independently perform FAST [15, 16], or under the supervision of a radiologist. There were no FAST examinations that were performed by other professionals. FAST was performed in a standardized manner by screening for free intra-abdominal fluid in the hepatorenal and splenorenal recesses, paracolic gutters, and rectovesical or rectouterine pouch, using a Zonare ZS3 Premium Ultrasound System with a C6-2 convex array transducer (Zonare Medical Systems). FAST examinations were classified as either positive (i.e., presence of free intra-abdominal fluid), negative (i.e., absence of free intra-abdominal fluid), or inconclusive (if one or more of the above-mentioned compartments could not be adequately visualized). Free fluid isolated to the rectouterine pouch in female patients of reproductive age and a small amount of isolated free fluid in the pelvis in children were considered physiologic [17–19]. Because all FAST examinations were prospectively performed, FAST interpreters were blinded to patient outcome.

### CT

Based on history taking and clinical examination, the (trauma) surgeon decides whether the suspicion of abdominal injury is sufficiently high to perform additional abdominal CT after FAST, unless the patient is too hemodynamically unstable for CT. CT was performed using a Siemens SOMATOM Definition Edge system (Siemens Healthineers) with a constant tube potential of 100 or 120 kV and an automatic adjustment of mAs in the z-direction. CT scans were interpreted by or under the supervision of radiologists using a Carestream Vue PACS version 11.4.1.1102 workstation (Carestream Health). Because all CT examinations were prospectively performed, CT interpreters knew the FAST result, but were blinded to patient outcome. CT was considered positive for abdominal injury if damage to any organ or pathologic free intra-abdominal fluid was reported. In patients with multiphase contrast-enhanced CT, the presence of contrast extravasation was also reported.

### Statistical analysis

Clinical variables of patients with traumatic abdominal injury and a false-negative FAST were compared to those of patients with traumatic abdominal injury and a true-positive FAST, using the unpaired *t* test for Gaussian data, the Mann-Whitney test for non-Gaussian data, and the Fisher test for dichotomous and ordinal data. Univariate logistic regression analyses were performed to determine the association of patient outcome (defined as the need for an interventional radiologic procedure, laparotomy, or death due to abdominal injury) with clinical variables, FAST result, presence of pathologic free intra-abdominal fluid on CT, and presence of contrast extravasation on CT. Variables that were significant on univariate analysis were subjected to multivariate analysis. Although CT is widely available in the developed world, this is not the case in developing countries [20–22]. Therefore, a multivariate analysis was also run without CT variables, provided one of them was significant on univariate analysis. *p* values less than 0.05 were considered statistically significant. Statistical analyses were executed using IBM Statistical Package for the Social Sciences (SPSS) version 26 (SPSS, Chicago, IL, USA).

## Results

### Patients

**Fig. 1:**
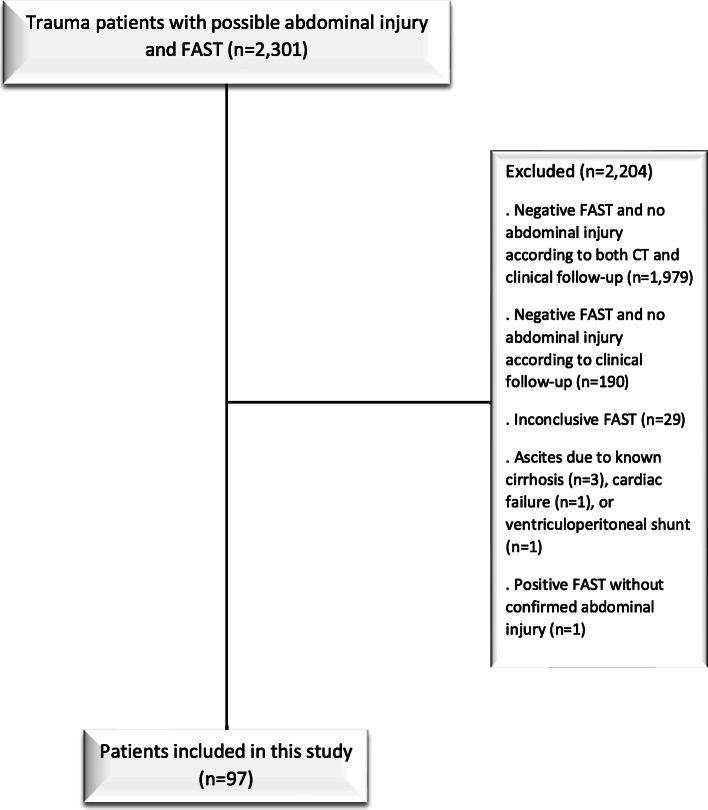
Flowchart of patient inclusion. Out of all 2301 consecutive patients who underwent FAST within a 26-month period at our institution, 314 underwent abdominal CT. Of 97 patients who were included in this study, 96 underwent abdominal CT, while 1 was not sufficiently hemodynamically stable to undergo abdominal CT, and this patient underwent immediate subsequent surgery. All other 96 patients with confirmed abdominal injury who were included in this study underwent abdominal CT after FAST. Also note that when considering the entire group of patients who undergoes FAST, patients with a negative FAST will have a better outcome than those with a positive FAST, regardless of whether they actually have abdominal injury or not. However, before doing this study, it was unclear if FAST has any value in predicting outcome in patients who actually do have abdominal injury. This was the reason of excluding patients without confirmed abdominal injury

A total of 2301 consecutive trauma patients underwent FAST. After applying the exclusion criteria, 97 patients with confirmed abdominal injury were available for further analysis (Fig. 1). Median patient age ± interquartile range was 34.0 ± 34.0 years, and male/female distribution was 59/38. FAST was false-negative in 40 patients (41.2%) and true-positive in 57 patients (58.8%). Ninety-four cases were due to blunt trauma, two cases were due to penetrating injury, and in 1 case the type of trauma was unknown. Characteristics of included patients are summarized in Table 1. Patients with a false-negative FAST were significantly older (*p* = 0.005), more frequently had higher hemoglobin levels (*p* = 0.015), and had a longer time interval between FAST and abdominal CT (*p* = 0.029) than patients with a true-positive FAST. Other clinical variables (including presence or absence of code red level of urgency and extra-abdominal injury) were not significantly different between the two groups (Table 1).

**Table 1:** Characteristics of the 97 included patients

Variable	All patients (*n* = 97)	Patients with false-negative FAST (*n* = 40)	Patients with true-positive FAST (*n* = 57)	*P* value
Age (years), median ± IQR (range)	34.0 ± 34.0 (6.0–89.0)	44.5 ± 46.0 (10.0–89.0)	27.0 ± 29.0 (6.0–86.0)	*0.005*
Male gender, *n* (%)	59 (60.8)	21 (52.5)	38 (66.7)	0.206
Blunt abdominal trauma, *n* (%)^a^	94 (96.9)	39 (97.5)	55 (96.5)	0.312
Code red level of urgency, *n* (%)^b^	45 (45.9)	15 (37.5)	30 (52.6)	0.239
Low hemoglobin levels, *n* (%)^a^	54 (55.7)	16 (40.0)	38 (66.7)	*0.015*
Heart rate (beats per minute), mean ± SD (range)	93 ± 20 (50–155)	94 ± 19 (57–155)	92 ± 21 (50–133)	0.748
Systolic blood pressure (mmHg), mean ± SD (range)	116 ± 22 (66–166)^c^	121 ± 20 (85–160)^d^	112 ± 23 (66–166)^e^	0.498
Class 1 shock index (no shock), *n* (%)	15 (15.5)	7 (17.5)	8 (14.0)	0.195
Class 2 shock index (mild shock), *n* (%)	57 (58.8)	24 (60)	33 (57.9)	
Class 3 shock index (moderate shock), *n* (%)	19 (19.6)	9 (22.5)	10 (17.5)	
Class 4 shock index (severe shock), *n* (%)	6 (6.2)	-	6 (10.5)	
Extra-abdominal injury, *n* (%)	75 (77.3)	33 (82.5)	42 (73.7)	0.337
Time between FAST and abdominal CT (minutes), median ± IQR (range)^b^	28.5 ± 31 (7–4337)	31.5 ± 84 (7–4337)	25 ± 23.5 (7–270)	*0.029*
Hospitalization (days), median ± IQR (range)	12.0 ± 17.0 (0.0–70.0)	12.0 ± 16 (0.0–70.0)	12.0 ± 19.0 (0.0–59.0)	0.949

Significant *p* values (*p* < 0.05) are shown in italics

*IQR* interquartile range

^a^One case missing for this variable

^b^Six cases missing for this variable

^b^Thirteen cases has a systolic blood pressure below 90 mmHg

^d^Three cases has a systolic blood pressure below 90 mmHg

^e^Ten cases has a systolic blood pressure below 90 mmHg

### Reference standard for traumatic abdominal injury

Abdominal injury was confirmed on CT in 88 patients, on both CT and surgery in 8 patients, and on surgery in 1 patient. Of the 96 patients who underwent CT, scanning was performed in both the arterial and portal venous phase (*n* = 76), in both the arterial, portal venous, and excretory phase (*n* = 12), in the portal venous phase only (*n* = 5), in the arterial phase only (*n* = 1), in both the portal venous and excretory phase (*n* = 1), and without intravenous contrast medium (*n* = 1). In the patient who underwent CT without intravenous contrast medium, abdominal injury was confirmed by the presence of pathological free fluid that could not be explained by any other cause. Table 2 summarizes the abdominal injuries with corresponding FAST results in the 97 included patients.

**Table 2:** Summary of abdominal injuries and FAST results in the 97 included patients

Abdominal injury location^a^	No. (%)	False-negative FAST (No.)	True-positive FAST (No.)
Spleen only	29 (29.9)	13	16
Liver only	14 (14.4)	1	13
Kidney only	7 (7.2)	5	2
Bowel only	6 (6.2)	3	3
Liver and spleen	5 (5.2)	1	4
Kidney and spleen	4 (4.1)	1	3
Pancreas only	3 (3.1)	1	2
Adrenal gland and liver	2 (2.1)	0	2
Kidney and liver	2 (2.1)	0	2
Renal artery only	2 (2.1)	2	0
Superior mesenteric artery only	2 (2.1)	1	1
Adrenal gland and kidney	1 (1.0)	1	0
Adrenal gland, kidney, and liver	1 (1.0)	1	0
Adrenal gland, kidney, and spleen	1 (1.0)	1	0
Adrenal gland, liver, and spleen	1 (1.0)	1	0
Bowel and liver	1 (1.0)	0	1
Bowel, iliac vein, and ureter	1 (1.0)	0	1
Bowel, pancreas, and spleen	1 (1.0)	0	1
Inferior mesenteric artery only	1 (1.0)	0	1
Kidney, psoas muscle, and spleen	1 (1.0)	0	1
Renal artery and spleen	1 (1.0)	0	1
Vascular injury not further specified	1 (1.0)	0	1
Pathologic free intra-abdominal fluid without detectable organ injury at CT or surgery	10 (10.3)	8	2

^a^Ninety-four cases were due to blunt trauma, two cases were due to penetrating injury (one case with kidney and liver injury and one case with bowel, iliac vein, and ureteral injury), and in 1 case the type of trauma was unknown

### Patient outcome

Twenty-two of 97 patients (22.7%) had an unfavorable outcome due to abdominal injury, of whom 18 with a true-positive FAST and 4 with a false-negative FAST (Table 3).

**Table 3:** FAST results in the 22 patients with an unfavorable outcome

Unfavorable event description	False-negative FAST (No.)	True-positive FAST (No.)
Interventional radiologic procedure to treat abdominal hemorrhage	2	9
Interventional radiologic procedure to treat abdominal hemorrhage and eventually death due to abdominal injury		1
Laparotomy	2	5
Laparotomy and eventually death due to abdominal injury		1
Interventional radiologic procedure to treat abdominal hemorrhage, laparotomy, and eventually due to abdominal injury		1
Death due to abdominal injury		1

### Association between FAST result and other variables with patient outcome

On univariate analysis, a false-negative FAST (OR, 0.24; *p* = 0.017) and a higher systolic blood pressure (OR, 0.97 per mmHg increase; *p* = 0.034) were significantly associated with a favorable outcome, whereas contrast extravasation on CT (OR, 7.17; *p* = 0.001) and shock index classification (OR, 1.89 for each higher class; *p* = 0.046) were significantly associated with an unfavorable outcome (Table 4).

**Table 4:** Association of variables with an unfavorable outcome in univariate analysis, multivariate analysis, and multivariate analysis without CT variables

Variable	Univariate OR (95% CI)	*P* value	Multivariate OR (95% CI)	*P* value	Multivariate OR without CT variables (95% CI)	*P* value
Age	1.01 (0.99–1.03)^d^	0.183				
Male gender	0.91 (0.34–2.39)	0.850				
Code red level of urgency^a^	2.14 (0.79–5.77)					
Blunt abdominal trauma	0.28 (0.01–4.79)	0.385				
Extra-abdominal injury	0.99 (0.32–3.09)	0.995				
Low hemoglobin level^b^	1.48 (0.55–3.97)^e^	0.428				
Heart rate	1.00 (0.98–1.028)^f^	0.723				
Systolic blood pressure	0.97 (0.95–0.99)^g^	*0.034*	0.98 (0.96–1.01)^g^	0.270	0.98 (0.95–1.00)^g^	0.090
Shock index classification	1.89 (1.01–3.55)^h^	*0.046*				
False-negative FAST	0.24 (0.07–0.77)	*0.017*	0.44 (0.12–1.57)	0.208	0.28 (0.08–0.93)	*0.038*
Contrast extravasation on CT^c^	7.17 (2.24–22.97)	*0.001*	4.64 (1.33–16.15)	*0.016*		
Free intra-abdominal fluid on CT	1.53 (0.31–7.61)	0.597				

Significant *p* values (*p* < 0.05) are shown in italics

*CI* confidence interval, *OR* odds ratio

^a^Six cases missing for this variable

^b^One case missing for this variable

^c^Two cases missing for this variable

^d^Per year increase

^e^Per mmol/L increase

^f^Per beat per minute increase

^g^Per mmHg increase

^h^For each higher class

Because systolic blood pressure was more significant on univariate analysis than shock index classification, and both metrics are correlated, shock index classification was not entered into the multivariate model. On multivariate analysis, only contrast extravasation on CT remained significantly associated with an unfavorable outcome (OR, 4.64; *p* = 0.016), whereas FAST result and systolic blood pressure had no significant independent association with outcome (Table 4).

When excluding contrast extravasation on CT from multivariate analysis, only a false-negative FAST result was predictive of a favorable outcome (OR, 0.28; *p* = 0.038), whereas systolic blood pressure had no significant independent association with outcome (Table 4).

## Discussion

This study investigated the value of a false-negative FAST in predicting outcome in trauma patients with confirmed abdominal injury. Our results show that a false-negative FAST is suggestive of a better outcome than a true-positive FAST in confirmed traumatic abdominal injury. This finding seems plausible, because traumatic abdominal injury without any detectable free intra-abdominal fluid on FAST likely indicates a lower risk of clinically relevant organ damage and/or hemodynamic instability that would require invasive treatment and increase mortality risk. Of interest, the time between FAST and abdominal CT was significantly longer in patients with a false-negative FAST than in patients with a true-positive FAST. This may suggest that a low flow of or delayed bleeding may be responsible for false-negative FAST results. Importantly, however, a negative FAST may still miss patients at risk of an unfavorable outcome: 4 out of 40 patients (10%) without free intra-abdominal fluid on ultrasonography eventually required an interventional radiologic procedure (*n* = 2) or laparotomy (*n* = 2), although it should also be mentioned that there were no deaths due to abdominal injury in the group of patients with a negative FAST. Furthermore, FAST lost its significance on multivariate analysis, in which contrast extravasation on CT remained as the only significant predictor of an unfavorable outcome. Therefore, FAST cannot substitute for abdominal CT in patients with suspected traumatic abdominal injury, and adequacy of staff and CT scanner availability should be ensured for these patients.

Nevertheless, the predictive value of the FAST result may still be clinically useful. FAST outperformed basic vital measurements including heart rate, systolic blood pressure and shock index class in predicting an unfavorable outcome in patients with abdominal injury. This can be explained by the fact that FAST specifically provides information about the abdomen, whereas these basic vital measurements may be affected by extra-abdominal conditions. Therefore, the FAST result is an important parameter in triaging patients (i.e., deciding the order of patients) for subsequent abdominal CT. This may be useful when multiple trauma patients simultaneously present in the emergency department, and when available staff and CT systems are temporarily limited. Cases with a positive FAST may be prioritized for abdominal CT before those with a negative FAST when clinical presentation is otherwise similar. Furthermore, in hospitals in the developing world in which CT is unavailable [20–22], ultrasonography can be the only imaging tool for risk stratification and prognostication of patients with a high suspicion of abdominal injury. Note that abdominal injury can be suspected based on clinical grounds, a positive FAST, or the presence of hematuria, fracture of the lower ribs, lumbar spine, or pelvis (which can be visualized on conventional radiography) in patients with a negative FAST [23]. Finally, cases with a positive FAST may require more intense observation to detect and treat clinical deterioration in a timely manner, which may potentially improve outcome, but this has to be proven by future studies.

Over the past three decades, the treatment of abdominal injury has shifted from operative towards more non-surgical management strategies, particularly when the patient has no sign of internal bleeding or peritonitis [24–26]. In this context, it has become increasingly important to obtain predictors of an adverse outcome. Such predictors may identify patients who require more intense observation to detect and manage clinical deterioration in a timely manner and to provide patients prognostic information about their outcome. A previous study by Laselle et al. [11] included 332 consecutive patients who presented to an urban level I trauma center in the USA. All patients presented with blunt abdominal trauma, had a documented FAST, and pathologic free fluid as determined by CT, diagnostic peritoneal lavage, laparotomy, or autopsy. Laselle et al. [11] reported a false-negative FAST result to be not associated with mortality, prolonged intensive care unit length of stay, or total hospital length of stay. This can be explained by the fact that trauma patients frequently present with extra-abdominal injuries, which is a confounding factor in these analyses. Laselle et al. [11] also reported that patients with false-negative FAST results were substantially less likely to require therapeutic laparotomy with an OR of 0.31 (95% CI, 0.19–0.52). This finding is in line with the results of the present study. However, Laselle et al. [11] did not taken into account the need for an interventional radiologic procedure and death due to abdominal injury, and they did not include CT findings in their logistic regression analysis. Other studies on the association between FAST and outcome in patients with confirmed traumatic abdominal injury are currently lacking. The present study therefore provides unique data on the value of a false-negative FAST in predicting outcome related to abdominal injury, in terms of the need for a subsequent interventional radiologic procedure or laparotomy, and death.

The present study had some limitations. First, it was performed in a tertiary care center in Europe, where the majority of abdominal traumas results from a blunt mechanism and penetrating lesions are less frequent [2]. Only 2 of 97 cases (2.1%) suffered from penetrating trauma in the present study. The results may be different in other non-European countries in which the incidence of gunshot or stab wounds is higher. Second, the results of this study are only applicable to settings in which FAST is used as an upfront screening tool in all patients with possible abdominal injury, and not in institutions in which FAST is only performed in hemodynamically unstable patients and CT is done instead of FAST in hemodynamically stable patients [27]. Third, FAST was performed by 27 different radiology residents in this study, which may have introduced observer bias that could not be accounted for. However, this reflects clinical practice. Fourth, FAST cannot discriminate between free intra-abdominal fluid due to trauma and pre-existent ascites that may have numerous causes [10], and 5 of such cases (3 with cirrhosis, 1 with cardiac failure, and 1 with a ventriculoperitoneal shunt) had to be excluded from our study. This potential pitfall should be taken into account when performing FAST in trauma patients. Fifth, standard B-mode ultrasonography was used in this study, whereas newer techniques such as contrast-enhanced ultrasonography may have the potential to improve the detection of traumatic injury [28]. However, whether or not such an approach is feasible in trauma patients remains unclear. Sixth, the number of patients was too low to perform subgroup analyses according to the type of abdominal injury and to determine if healthcare providers can take the time to perform CT and avoid emergent laparotomy in patients with a negative FAST. Seventh, our study included 15 children (i.e., aged 17 years or younger) of whom 3 (20.0%) with a false-negative FAST and 82 adults (i.e., aged 18 years or older) of whom 37 (45.1%) with a false-negative FAST. Unfortunately, the number of cases was too low to statistically compare the outcome of children to that of adults with a false-negative FAST. Eighth, the decision to perform additional abdominal CT after FAST was based on the (trauma) surgeon’s clinical judgment, for which no clear criteria could be defined. Ninth, we did not have any dedicated software or other tools to reproducibly quantify the amount of free intra-abdominal fluid on CT, whereas this may be another variable potentially related to false-negative FAST results and favorable outcome [29]. Nevertheless, in resource-constrained regions where CT is not available (and the amount of free intra-abdominal fluid cannot be measured on CT), the FAST examination may be the only imaging tool on which management decisions are based. Tenth, only the presence of and not the severity of injuries was considered in the analysis of results.

In conclusion, trauma patients with confirmed abdominal injury and a false-negative FAST have a better outcome than those with a true-positive FAST. FAST may be valuable for risk stratification and prognostication in patients with a high suspicion of abdominal injury when CT has not been performed yet or when CT is not available.

## Data Availability

Available upon request to the authors

## Abbreviations

CI: Confidence interval
CT: Computed tomography
FAST: Focused abdominal sonography for trauma
IQR: Interquartile range
OR: Odds ratio
SPSS: Statistical Package for the Social Sciences
